# Is low birth weight associated with lower adiponectin levels? - A systematic review and meta-analysis

**DOI:** 10.1371/journal.pone.0335598

**Published:** 2025-12-02

**Authors:** Afnan Sabah Alhakeem, Aleyna Hasturk, Trine Witzner Hessel Lawaetz, Tue Helms Andersen, Charlotte Brøns, Allan Arthur Vaag

**Affiliations:** 1 Copenhagen University Hospital - Steno Diabetes Center Copenhagen, Herlev, Denmark; 2 Lund University Diabetes Center, Lund University, Malmö, Sweden; 3 Department of Endocrinology, Skåne University Hospital, Malmö, Sweden; Korea University - Seoul Campus: Korea University, KOREA, REPUBLIC OF

## Abstract

Individuals born with low birth weight are at increased risk of type 2 diabetes, which potentially may be attributed to immature adipose tissue development and reduced levels of the insulin-sensitizing adipokine, adiponectin. This systematic review and meta-analysis synthesize data from 67 studies, comprising over 8000 individuals across various age groups, to examine the relationship between circulating adiponectin levels and birth weight. The results revealed that individuals with low birth weight have significantly lower adiponectin levels compared to those born with normal birth weight (SMD = −0.46 μg/ml [95% CI: −0.57; −0.35], P < 0.0001). Moderate heterogeneity was observed (I^2 ^= 67%, P < 0.01), but sensitivity analysis and meta-regression did not identify specific factors driving this variation. Pooled Pearson correlation analysis indicated a moderate but statistically significant positive correlation between birth weight and adiponectin levels (correlation estimate = 0.31 [95% CI: 0.16; 0.46], P < 0.0001). These findings suggest that reduced adiponectin levels in low birth weight individuals may contribute to their elevated risk of type 2 diabetes, potentially offering new insights into the developmental origin of this disease.

## Introduction

The association between being born with a low birth weight (LBW) and later increased risk of developing type 2 diabetes (T2D) and associated cardiometabolic diseases has been firmly established [1–4]. The concept of developmental programming influenced by adverse environmental factors during early growth provides a conceptual framework to explain how immature development and age-dependent dysfunctions of multiple organs are involved in T2D development [5]. T2D is a multifactorial heterogeneous disease that involves a complex interplay between pancreatic beta-cells, systemic insulin resistance, and impaired adipose tissue expandability [6–8]. Notably, emerging evidence indicates that adverse fetal conditions associated with LBW can lead to epigenetic changes that affect genes involved in adipogenesis, resulting in immature development of the subcutaneous adipose tissue (SAT) [9]. This may disrupt the normal endocrine functions of the tissue, including the secretion of adiponectin. Adiponectin is considered to play a central role in enhancing insulin sensitivity [10]. Notably, studies have reported reduced adiponectin levels in LBW subjects [8,11–13], which therefore may contribute to insulin resistance, ectopic fat deposition, metabolic dysfunction-associated steatotic liver disease (MASLD), and eventually overt T2D in these individuals [10,8,14]. Accordingly, reduced adiponectin levels may play a pivotal role in mediating the risk of developing T2D and associated cardiometabolic comorbidities in LBW subjects [15–17]. Therefore, we performed a systematic literature review and meta-analysis to examine whether LBW is associated with low circulating adiponectin levels when compared with normal birth weight (NBW).

## Methods

This systematic review was reported according to the Preferred Reporting Items for Systematic Reviews and Meta-Analysis (PRISMA) criteria [15]. The protocol was registered in the PROSPERO Database (registration ID: CRD42023461784).

### Search strategy

Potentially relevant studies were identified through a comprehensive search in MEDLINE, Embase, and Web of Science core collection on July 4, 2024. An information specialist (THA) conducted a systematic search with the concepts “adiponectin/adipokines” and “low birth weight”. Both concepts were searched using Medical Subject Headings (MeSH) and free text words. The search string was peer reviewed by another information specialist. Further, we evaluated it by testing whether 21 key articles within the field were among the search results. The search string was developed in MEDLINE and subsequently translated to the other databases. No limit on date, country, or language was applied. Details on the search strategy for each database can be found in S1 Table.

Citation tracking was performed on all articles included in the review to retrieve references and articles citing the included articles with the software tool Citationchaser [16].

### Eligibility criteria

Observational studies that provided data on birth weight and circulating adiponectin levels in healthy human participants across various age groups were included. Studies were included irrespective of whether they reported categorical comparisons between groups (i.e., LBW and/or high birth weight (HBW) versus NBW subjects) or adiponectin measurements across the entire spectrum of birth weights. We intended to adhere to the original studies’ criteria for birth weight categories, where small and large for gestational age (GA) were considered equivalent to low and high birth weight, respectively [17]. The operational definition of LBW was a birth weight below 2500 grams, irrespective of GA, and includes both preterm infants with appropriate growth (>37 completed weeks of gestation) as well as term and preterm growth-restricted infants (<10th centile of weight for gestational age and sex), commonly referred to as small for gestational age (SGA) [18,19]. We incorporated both absolute weight (grams) and percentiles in defining LBW to reflect the equivalence between LBW and SGA, as absolute weight typically defines LBW while percentiles are used for SGA. Importantly, some studies use a threshold of less than 2 standard deviations (SD) or the 10th percentile to define this category. HBW was categorized as either a birth weight above the 90th percentile or over 4000 grams. For control groups, birth weight was defined as between the 10th and 90th percentiles, within ± 1 SD, or within the range of 2500–4000 grams. Studies reporting birth weight in grams according to clinical definitions all accounted for GA by including full-term infants (GA = 37–41 weeks) [20–24]. Subjects from the studies were divided into different age groups, including neonates (birth to 28 days) [25], infants (28 days to 12 months) [25], children (1 year to 17 years) [26], and adults (18 years and older) [26], reflecting the aim to explore adiponectin levels across various age groups. Adiponectin concentrations had to be reported in either plasma, serum, or cord blood. Studies meeting the following criteria were excluded: a) animal studies, b) clinical trials, c) populations with acute and/or chronic diseases, including diabetes, d) studies with fewer than 12 subjects, and e) missing information on the applied adiponectin assay. All studies meeting the inclusion criteria and providing extractable data were considered for inclusion in this analysis. The specific inclusion and exclusion criteria applied in the study selection process are summarized in **Table 1**.

**Table 1:** Inclusion and exclusion criteria.

Criteria	Inclusion	Exclusion
Population	Human studies investigating individuals with LBW compared to NBW	Animal studies or in vitro/ex vivo studies
Exposure	LBW, IUGR, SGA, or impaired fetal growth	Studies without a clear definition of LBW or fetal growth
Outcome	Circulating adiponectin levels measured in blood, plasma, or serum	Studies not reporting adiponectin levels
Study design	Cohort studies, case-control studies, and cross-sectional studies	Reviews, commentaries, conference abstracts, editorials, and case-report studies
Language	English, Arabic, Turkish, Danish, Swedish, and Norwegian	Other languages
Publication type	Peer-reviewed journal articles	Preprint or unpublished studies
Data availability	Studies providing mean adiponectin levels, SDs, SEs, or CIs	Studies lacking numerical adiponectin levels

LBW, *low birth weight;* NBW, *normal birth weight;* IUGR, *intrauterine growth restriction;* SGA, *small for gestational age;* SD, *standard deviation;* SE, *standard error;* CI, *confidence interval.*

### Study selection and data extraction

Records found in the database search were uploaded to EPPI reviewer 6 [27], and duplicates were removed. Two independent reviewers (ASA, AH) screened the titles and abstracts of all retrieved studies. The full text of potentially eligible studies was then screened by the same reviewers according to the inclusion criteria. Data were independently extracted by each reviewer and subsequently compared to ensure accuracy and consistency in data extraction. Information on authorship, publication year, country, study design, survey period, sample size, age, mean or median birth weight, mean or median adiponectin levels, assay type, and blood sample type was extracted. Any discrepancy between reviewers was resolved through discussion.

Articles in languages not understood by the review team (excluding English, Arabic, Turkish, Danish, Swedish, and Norwegian) that were deemed eligible by title and abstract were not included in the synthesis but have been listed for others to analyze (S2 Table).

### Quality assessment

The JBI critical appraisal tool [28–30] was applied to evaluate study quality. Each study underwent assessment regarding the selection of study population, description of study settings, exposure assessment, outcome assessment, identification of confounders, strategies for confounder adjustment, and appropriate statistical analysis. The two reviewers independently evaluated each study, and through discussion, a final score indicating either low, unclear, or high risk of bias was assigned. Of the included studies, a total of 33 were rated as low risk of bias, 24 had an unclear risk of bias, and 10 were rated as high risk of bias. A more detailed quality assessment is described in S1-S3 Figs. The most important limitations pertaining to the quality of the original reports were inadequate adjustment for confounders (29 studies), lack of clear description of the study population (4 studies), and reliance on self-reported birth weight information rather than register-based data (2 studies). Conversely, high-quality studies tended to use well-defined inclusion criteria and robust exposure and outcome assessment methods.

### Statistical analysis

The standardized mean difference (SMD) and corresponding 95% confidence interval (CI) were used to measure the difference in adiponectin levels between LBW and NBW subjects. Studies providing birth weight and adiponectin data as mean and SD were incorporated in the meta-analysis. In cases where a standard error of a mean (SEM) was given, it was converted to SD by multiplying SEM by the square root of the sample size [31]. The overall effect estimate was determined through the random effects model, where heterogeneity was assessed through Cochran’s Q and I^2^ statistics. Values below 25% indicate low heterogeneity, 25–75% moderate heterogeneity, and above 75% high heterogeneity. To explore the sources of heterogeneity, sensitivity analysis and meta-regression were conducted considering the following factors: age category, risk of bias, and sample type.

An influence analysis was performed to further explore the between-study heterogeneity by excluding studies one by one to identify potential studies contributing to distortions of the effect estimate. Robustness of the meta-analysis was explored using a GOSH Plot analysis to identify studies with high impact on heterogeneity. Publication bias was assessed through visual inspection of the funnel plot and Egger’s test, with a P < 0.05 indicating significant publication bias.

All statistical analyses were performed using R Software (version 4.3.0) with the metafor, dmetar, and tidyverse packages [32–34]. P-value < 0.05 was considered statistically significant. The analysis script is available at the following repository: https://github.com/TT2DR-StenoDiabetesCenterCopenhagen/Systematic_review_and_meta-analysis_adiponectin.git.

## Results

### Search results

**Fig 1:**
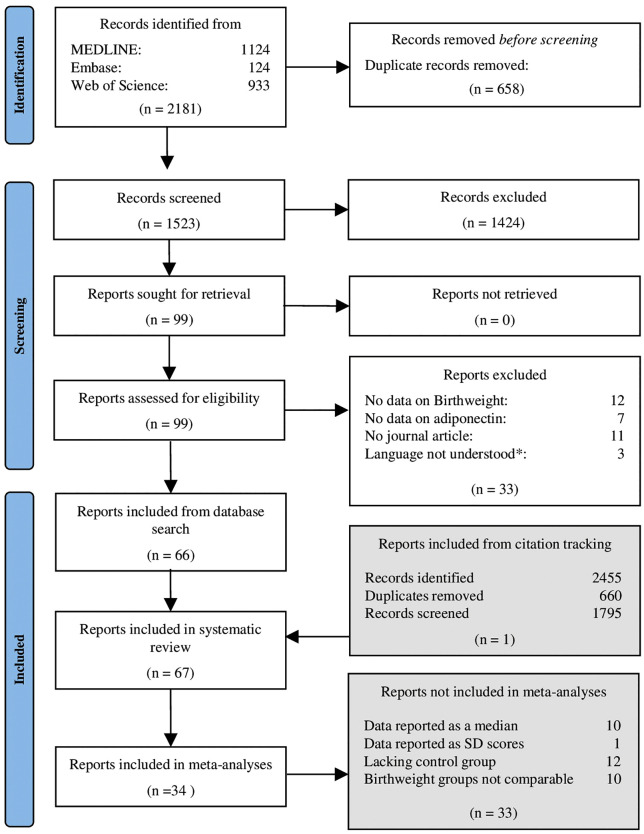
Flow diagram of the selection process. *Full-text articles only available in languages other than English, Danish, Swedish, Norwegian, Turkish, and Arabic.

A total of 2181 studies were identified through a systematic search in MEDLINE, Embase, and Web of Science from inception to July 4, 2024. After removing duplicates, 1523 studies were screened by title and abstract yielding a total of 99 eligible studies. Evaluating the full text of these studies resulted in 66 studies meeting the inclusion criteria. Citation tracking retrieved 2455 additional studies of which 660 were duplicates. One additional study from the citation tracking was deemed relevant, and a total of 67 studies were included in the systematic review (**Fig 1**).

### Study characteristics and quality assessment

**Table 2** displays the comprehensive characteristics of the studies, including 8204 individuals in the systematic review. Most studies categorized subjects based on LBW (n = 27) or HBW (n = 17), while others were stratified by maternal gestational diabetes (n = 6) or maternal smoking status (n = 2). Additionally, a few studies reported adiponectin levels in preterm (n = 5) or twin births (n = 3), where these subjects were grouped into their respective birth weight categories. Only observational studies were included, hereof 43 cross-sectional, 14 case-control, and 10 cohort studies. Information on both periods of data collection and population origins was documented. Adiponectin levels were measured in blood samples (n = 30), cord blood (n = 36), and one in a dried blood spot sample [35] and analyzed by enzyme-linked immunosorbent assay (ELISA, n = 47), radioimmunoassay (RIA, n = 18), multiplex assay (n = 1), or by immunoassay (n = 1). As for the specific molecular form of adiponectin, most studies reported total adiponectin levels. The studies were categorized according to the age groups of the subjects, among them, 28 studies focused on neonates, 19 on infants, 18 on children, and 2 studies on adults.

**Table 2:** Study characteristics.

1^st^ Author, year	Survey period	Origin of population	Study design	Sample size (n)	Age	Population description	Adiponectin			Birth weight category
							Assay Type	Sample	Molecular weight	
Sivan (2003)	–	Israel	Cross-sectional	67	Neonates	Neonates born either SGA or LA, and at term (GA ≥ weeks) or near term (GA between 34–36 weeks)	RIA	Cord blood, serum	**Total adiponectin	No categories
Chan (2004)	–	Taiwan	Cross-sectional	84	Neonates	Neonates from healthy mothers	RIA	Cord blood, serum	**Total adiponectin	No categories
Kamoda (2004)	–	Japan	Cross-sectional	62	Neonates	Neonates born either SGA or AGA	RIA	Cord blood, serum	Total adiponectin	AGA: > −2 to <+2 SD SGA: ≤ −2 SD
Kotani (2004)	–	Japan	Cohort	59	Neonates	Healthy neonates born at term (GA = 37–41 weeks)	Elisa	Cord blood, serum	**Total adiponectin	No categories
Lopez-Bermejo (2004)	–	Spain	Cross-sectional	69	Children	Prepubertal children (2.5–8.5 years) and prepubertal children (3–9 years)	Elisa	Venous blood, serum	**Total adiponectin	AGA: > 25^th^ percentile SGA: < 10^th^ percentile
Pardo (2004)	–	Brazil	Cross-sectional	132	Neonates	Healthy neonates born at term (GA = 35–42 weeks)	RIA	*Cord blood	**Total adiponectin	No categories
Tsai (2004)	2001-2002	Taiwan	Cross-sectional	226	Neonates	Healthy neonates born either AGA or LGA, and at term (GA ≥ 37 weeks)	RIA	Cord blood, plasma	**Total adiponectin	AGA: 10^th^-90^th^ percentile LGA: > 90^th^ percentile
Mazaki-Tovi (2005)	–	Israel	Cross-sectional	81	Neonates	Healthy neonates born either LGA or AGA at term (GA = 37–41 weeks)	RIA	*Cord blood	Total adiponectin	AGA: 10^th^-90^th^ percentile LGA: > 90^th^ percentile
Jaquet (2006)	–	France	Cross-sectional	1059	Adults	Young adults (18.5–27 years) born SGA and adults (18.5–27 years) born AGA	RIA	Venous blood, serum	**Total adiponectin	AGA: 25^th^ −75^th^ percentile SGA: < 10^th^ percentile
Kim (2006)	2003	Korea	Cross-sectional	152	Children	Pubertal children (12–15 years) born AGA	Elisa	Venous blood, serum	**Total adiponectin	NBW: 2500g-4200g LBW: < 2500g Birth records and maternal recall
Martinez-Cordero (2006)	–	Mexico	Cross-sectional	100	Infants	Healthy SGA and AGA infants born at term (GA = 37–41 weeks)	RIA	*Cord blood	**Total adiponectin	AGA: > 2500g and <4000g SGA: ≤ 2500g
Evagelidou (2007)	1996-1997	Greece	Case-control	70	Children	Prepubertal children (6–8 years) born either AGA or SGA	Elisa	Venous blood, serum	**Total adiponectin	AGA 10^th^ −90^th^ percentile SGA: < 10^th^ percentile
Giapros (2007)	–	Greece	Case-control	81	Children	Non-obese prepubertal children (5.5–7 years) born either LGA or AGA	Elisa	Venous blood, serum	**Total adiponectin	AGA: 10^th^ −90^th^ percentile LGA: > 90^th^ percentile
Inami (2007)	2004-2005	Japan	Cohort	63	Neonates	Healthy neonates	Elisa	Cord blood, serum	**Total adiponectin	No categories
Kamoda (2007)	–	Japan	Cross-sectional	36	Children	Prepubertal children born either SGA or AGA	Elisa	Venous blood, serum	**Total adiponectin	AGA: > 10^th^ percentile SGA: < 10^th^ percentile
Klamer (2007)	–	Denmark	Case-control	60	Infants	Premature SGA and premature AGA (GA < 32 weeks), and mature SGA and mature AGA (GA = 37–42 weeks)	Luminex xMAP	DBSS	**Total adiponectin	AGA: not defined SGA: < −2 SD
Siahanidou (2007)	–	Greece	Cross-sectional	62	Infants	Preterm infants (mean (SD) GA = 32 (2.1) weeks) and full-term infants (GA > 37 weeks)	RIA	Venous blood, serum	**Total adiponectin	Full term: not defined Preterm: 28–36 weeks of gestation
Takaya (2007)	–	Japan	Cross-sectional	65	Neonates	Neonates born either SGA or AGA	Elisa	Cord blood, plasma	Total adiponectin	AGA: ≥ 10^th^ percentile SGA: < −1.5 SD
Zare (2007)	2005-2006	Iran	Cross-sectional	72	Neonates	Healthy neonates born AGA at term (GA = 37–41 weeks)	Elisa	Cord blood, serum	**Total adiponectin	No categories
Gohlke (2008)	–	Germany	Cross-sectional	62	Neonates	Healthy neonates from women with monochorionic monozygotic twin pregnancies and TTTS	RIA	Cord blood, serum	**Total adiponectin	No definition of birth weight categories
Ibanez (2008)	–	Spain	Cross-sectional	94	Children	Children (6 years) born SGA matched with children (6 years) born AGA	Elisa	Venous blood, serum	HMW adiponectin	AGA: −1 to +1 SD SGA: < −2 SD
Kyriakakou (2008)	–	Greece	Case-control	40	Neonates	Singleton births of AGA or asymmetric IUGR infants born at term (GA = 37–41 weeks)	RIA	Cord blood, serum	**Total adiponectin	AGA: not defined SGA: < 3^rd^ percentile
Cekmez (2009)	2006-2007	Turkey	Cross-sectional	100	Infants	LGA and AGA infants	RIA	*Cord blood	**Total adiponectin	AGA: 10^th^ −90^th^ percentile or 2500g-4000g LGA: > 90^th^ or>4000g
Challa (2009)	1997-1998	Greece	Cross-sectional	57	Children	Non-obese prepubertal children (4–10 years) born either SGA or AGA	Elisa	Venous blood, serum	**Total adiponectin	AGA 10^th^-90^th^ percentile SGA: < 10^th^ percentile
Darendeliler (2009)	–	Turkey	Case-control	84	Children	Prepubertal non-obese children born LGA or AGA	RIA	*Venous blood	**Total adiponectin	AGA: 10^th^ −90^th^ percentile LGA: > 90^th^ percentile
Ibanez (2009)	2008	Spain	Cross-sectional	24	Children	Prepubertal children (mean age 7.5 years) born SGA or AGA, and SGA children with spontaneous catch-up growth	Elisa	Venous blood, serum	HMW adiponectin	AGA: −1 to +1 SD SGA: < −2 SD
Laudes (2009)	–	Germany	Cross-sectional	40	Neonates	Healthy neonates from mothers with GDM, pregnancy-associated hypertension, and preeclampsia	Elisa	Cord blood, serum	**Total adiponectin	No categories
Mazaki-Tovi (2009)	–	Israel	Cross-sectional	58	Neonates	Mothers with dichorionic twin pregnancies.	RIA	*Cord blood	**Total adiponectin	No categories
Nakano (2009)	–	Japan	Cross-sectional	52	Infants	AGA infants born at term (GA between 37–41 weeks)	Elisa	Cord blood, serum	**Total adiponectin	No categories
Dundar (2010)	–	Turkey	Cross-sectional	25	Infants	Infants from healthy mothers with natural healthy vaginal birth	Elisa	*Cord blood	**Total adiponectin	No categories
Mohamed (2010)	2007	Egypt	Case-control	60	Neonates	Macrosomic neonates to diabetic mothers, non-macrosomic neonates to diabetic mothers, and non-macrosomic neonates to non-diabetic mothers. All neonates were full-term (GA ≥ 37 weeks)	Elisa	Cord blood, serum	**Total adiponectin	AGA: 2500g-4000g LGA: > 4000g
Nishihara (2010)	2004-2006	Japan	Cohort	83	Infants & children	Healthy infants and preschoolers (< 6 years of age)	Elisa	Blood, serum	**Total adiponectin	No categories
Wang (2010)	2007-2008	China	Cross-sectional	100	Neonates	Neonates born with FGR, AGA, or macrosomia	Elisa	Cord blood, serum	**Total adiponectin	AGA: 2500g-4000g LGA: > 4000g SGA: < 2500g
Ballesteros (2011)	–	Spain	Case-control	212	Infants	Infants of mothers with GDM and infants of mothers with NGT	Elisa	*Cord blood	Total adiponectin	No categories
Cekmez (2011)	2007-2008	Turkey	Cross-sectional	74	Infants	LGA infants of GDM mothers and AGA infants of mothers with NGTT	RIA	Cord blood, plasma	**Total adiponectin	AGA: 10^th^ −90^th^ percentile or 2500g-4000g LGA: > 90^th^ or>4000g
Challa (2011)	–	Greece	Cross-sectional	98	Children	Non-obese prepubertal (5.5–8 years) children born either LGA or AGA	Elisa	Venous blood, serum	**Total adiponectin	AGA 10^th^ −90^th^ percentile SGA: < 10^th^ percentile
Meral (2011)	2007-2008	Turkey	Cross-sectional	60	Neonates	Healthy neonates born either SGA, AGA, or LGA at term (GA = 37–41 weeks)	Elisa	Blood, serum	**Total adiponectin	AGA: 10^th^ −90^th^ percentile or 2500g-4000g SGA: < 10^th^ percentile or <2500g LGA: > 90^th^ percentile or>4000g
Saito (2011)	–	Japan	Cohort	49	Infants	Infants born either SGA or AGA. All born prematurely	Elisa	Heel-prick, serum	**Total adiponectin	AGA: > −2 to <+2 SD SGA: < −2 SD
Palcevska-Kocevska (2012)	–	Macedonia	Cross-sectional	110	Neonates	Neonates born SGA, AGA or LGA at term (GA = 37–42 weeks)	Elisa	Venous blood	**Total adiponectin	AGA: 10^th^-90^th^ percentile SGA: < 10^th^ percentile LGA: > 90^th^ percentile
Cekmez (2013)	2007-2008	Turkey	Cross-sectional	81	Infants	Term, preterm, and ELBW neonates born to healthy mothers.	Elisa	Cord blood, serum	**Total adiponectin	AGA 10^th^ −90^th^ percentile SGA: < 10^th^ percentile
Hill (2013)	2007-2010	Canada	Cross-sectional	95	Children	Pubertal obese children (10–16 years)	Elisa	Venous blood, serum	HMW adiponectin	No categories
Strufaldi (2013)	–	Brazil	Case-control	180	Children	Prepubertal/pubertal children (6–11 years) born either with a LBW or with a NBW at term (GA > 37 weeks)	Elisa	Venous blood, serum	**Total adiponectin	NBW: ≥ 3000g- ≤ 4000g LBW: ≤ 2500g
Chelchowska (2014)	–	Poland	Cross-sectional	85	Neonates	Healthy infants from non-smoking and smoking mothers	Elisa	Cord blood, serum	Total adiponectin	No categories
Cianfarani (2014)	–	Italy	Cross-sectional	92	Children	Obese AGA, short-AGA and SGA children (5–12 years)	RIA	*Venous blood	**Total adiponectin	AGA 10^th^ −90^th^ percentile SGA: < 3^rd^ percentile
Terrazzan (2014)	–	Brazil	Cross-sectional	127	Infants	VLBW children born premature (GA < 32 weeks) and full-term (GA ≥ 37 weeks) neonates	Elisa	Cord blood, plasma	**Total adiponectin	Full term: ≥ 37 weeks of gestation Preterm: < 32 weeks of gestation and <1500g
Zheng (2014)	2009-2011	China	Cross-sectional	226	Neonates	Healthy full-term (GA = 37–41 weeks) neonates	Elisa	Cord blood, plasma	HMW adiponectin	No categories
Hansen-Pupp (2015)	2005-2007	Sweden	Cohort	49	Infants	Preterm infants (GA < 31 weeks)	Elisa	Cord blood, serum	**Total adiponectin	AGA: not defined SGA < −2 SD
Huang (2015)	–	China	Case-control	30	Children	Prepubertal short children (3.5–9.5 years) born SGA and prepubertal short children (4.5–8.5 years) born AGA	Elisa	Venous blood, serum	**Total adiponectin	AGA: ≤ +2 SDS SGA: < −2 SDS
Fonseca (2016)	2005-2006	Italy	Cross-sectional	392	Neonates	Neonates born with low, normal, or high BW	Elisa	*Cord blood	**Total adiponectin	No categories
Lausten-Thomsen (2016)	–	Denmark	Case-control	60	Infants	Healthy neonates (GA > 36 weeks) born either LGA or AGA	Elisa	Cord blood, serum	**Total adiponectin	AGA: not defined LGA: < 90^th^ percentile
Stansfield (2016)	2001-2005	USA	Cross-sectional	575	Children	Children (14–18 years)	Elisa	*Venous blood	**Total adiponectin	No definition of birth weight categories
Aramesh (2017)	2014-2015	Iran	Case-control	104	Infants	Infants of mothers with GDM and infants of mothers with NGT	Elisa	Cord blood, serum	**Total adiponectin	No categories
Chen (2017)	–	China	Cohort	51	Neonates	Neonates of mothers with GDM	RIA	Cord blood, serum	**Total adiponectin	No categories
Milenkovic (2017)	2013-2016	Serbia	Cross-sectional	48	Neonates	Discordant twins and concordant twins born AGA at term (GA = 32–40 weeks)	RIA	Venous blood, serum	**Total adiponectin	No categories
Mohamed (2017)	2016-2017	Egypt	Cross-sectional	120	Neonates	Neonates born SGA, AGA, or LGA at term (GA ≥ 37 weeks).	Elisa	Cord blood, serum	**Total adiponectin	AGA: 10^th^-90^th^ percentile SGA: < 10^th^ percentile LGA: > 90^th^ percentile
Dong (2018)	2010-2012	Canada	Case-control	212	Infants	LGA infants matched with OGA controls	Elisa	Cord blood, serum	**Total adiponectin	AGA: 25^th^ −75^th^ percentile LGA: > 90^th^ percentile
Leniz (2019)	2013-2015	Spain	Cohort	27	Infants	Healthy infants born SGA at term (GA = 33–41 weeks) followed up at 3, 12, 18, and 24 months	Elisa	Venous blood, serum	**Total adiponectin	No categories
Yalinbas (2019)	2011-2012	Turkey	Cross-sectional	96	Neonates	Healthy neonates born SGA, AGA, or LGA	Elisa	Cord blood, serum	**Total adiponectin	AGA: 10^th^ −90^th^ percentile SGA: < 10^th^ percentile LGA: > 90^th^ percentile
Chelchowska (2020)	2012-2014	Poland	Cross-sectional	78	Neonates	Healthy infants from non-smoking and smoking mothers	Elisa	Cord blood, serum	Total adiponectin	No categories
Simeoni (2020)	–	France	Cohort	188	Adults	Healthy adults (18–25 years) born at term (GA > 37 weeks) and AGA	Elisa	*Venous blood	**Total adiponectin	No categories
Han (2021)	2011	China	Cross-sectional	149	Neonates	Neonates from healthy mothers born either at term (GA = 37–41 weeks) or preterm (GA < 37 weeks)	Elisa	*Cord blood	**Total adiponectin	Term: 37–41 weeks of gestation Preterm: < 37 weeks of gestation
Huang (2022)	–	Canada	Case-control	210	Infants	Infants from singleton pregnancies followed up at birth, and at 3, 12 and 24 months	Elisa	*Venous blood	HMW and total adiponectin	AGA: 25^th^ −75^th^ percentile LGA: > 90^th^ percentile
Pippen (2022)	–	USA	Cohort	336	Children	LGA vs. AGA children (median age 7 years)	Immunoassay	Blood, plasma	**Total adiponectin	AGA: 10^th^ −90^th^ percentile LGA: > 90^th^ percentile
Santana-Meneses (2022)	2016-2017	Brazil	Cross-sectional	54	Infants	Infants (9–12 months) with LBW born at term (GA ≥ 34 weeks)	Elisa	Venous blood, serum	**Total adiponectin	AGA: not defined SGA: < 2500g
Yalinbas (2022)	2021	Turkey	Cross-sectional	195	Neonates	Healthy neonates born SGA, AGA, or LGA	Elisa	Venous blood, serum	**Total adiponectin	AGA: 10^th^ −90^th^ percentile or 2500g-4000g SGA: < 10^th^ percentile or <2500g LGA: > 90^th^ percentile or>4000g
Huang (2023)	2010-2012	Canada	Cohort	205	Infants	Infants born either SGA or OGA	Elisa	Venous blood, plasma	HMW and total adiponectin	AGA: 25^th^ −75^th^ percentile SGA: < 10^th^ percentile
Zamojska (2023)	–	Poland	Case-control	60	Children	Healthy non-obese prepubertal children (7–9 years) born SGA or AGA	Elisa	Venous blood, serum	**Total adiponectin	AGA: not defined SGA: < 10^th^ percentile

GDM, *gestational diabetes mellitus*; NGT, *normal glucose tolerance*; LGA, *large for gestational age*; AGA, *appropriate for gestational age*; ELBW, *extremely low birth weight*; SGA, *small for gestational age*; DBSS, *dried blood spot sample*; OGA, *optimal for gestational age;* TTTS, *twin-twin transfusion syndrome*; GA, *gestational age*; LBW, *low birth weight*; BL, *birth length*; BW, *birth weight*; *IUGR*, *intrauterine growth retardation*; FGR, *fetal growth restriction*; VLBW, *very low birth weight*;. -, *No report*.

* Not clarified if adiponectin is measured in plasma or serum. All venous blood samples were taken in the fasting state (between 5–12 hours).

**Not clarified which molecular weight of adiponectin is measured; we are assuming that the assays are for total adiponectin.

### Meta-analysis

Out of the 67 studies included in the systematic review, only 34 studies were eligible for inclusion in the meta-analysis. Eleven studies were not incorporated as they reported data solely as medians or SD scores, 12 studies lacked a control group, and 10 studies did not compare different birth weight categories, instead focusing on variables such as smoking or gestational diabetes. The objective of this study was to investigate the difference in adiponectin levels between LBW and NBW subjects, as analyzed in a total of 24 studies included in our review. Given the broad inclusion criteria for birth weight, a subset of the included studies also examined adiponectin levels in HBW subjects. These data were incorporated into a pre-defined exploratory analysis to gain further insight into the broader relationship between adiponectin and birth weight. It is important to note that the primary focus of the search was on LBW, and as such, we cannot be certain that all studies concerning HBW were included. Although this exploratory analysis offers valuable preliminary insights, we do not believe that this due to the number and quality of studies, establishes any definitive relationship between HBW and adiponectin levels. Well-designed studies with robust methodologies are needed to confirm this association.

### Main analysis

Data from the 24 studies examining adiponectin levels in LBW and NBW subjects were included in the meta-analysis, which showed that LBW subjects had significantly lower adiponectin levels compared to NBW subjects (SMD = −0.56 μg/ml [95% CI: −0.99; −0.14], P = 0.01). The studies were characterized by substantial heterogeneity (I^2 ^= 92%, P < 0.01), which was investigated through sensitivity analysis and meta-regression on variables such as age, blood sample type, and risk of bias, supplemented by visual assessment (S4-S6 Figs). However, none of these variables were able to elucidate the underlying reasons for the observed high heterogeneity, as the outcome did not achieve statistical significance (**Table 3**).

**Table 3:** SMD and associated heterogeneity in a sensitivity analysis of 24 studies comparing adiponectin levels in LBW individuals with NBW controls (including outliers^1^).

Characteristics	Data points	Summary SMD (95% CI)	P-value for effect interaction	I^2^ (95% CI)	P-value for heterogeneity
Age categories					
Neonates	13	−0.68 [−1.5021; 0.1389]	0.06	94%	<0.001
Infants	1	−0.56 [−1.1162; −0.0150]		–	–
Children	11	−0.48 [−0.9997; 0.0383]		87%	<0.001
Adults	1	−0.08 [−0.2072; 0.0347]		–	–
Blood sample type					
Cord	9	−0.71 [−1.9997; 0.5629]	0.68	96%	<0.001
Blood	17	−0.48 [−0.8094; −0.1638]		84%	<0.001
Risk of bias					
Low	11	−0.61 [−1.0663; −0.1664]	<0.001	87%	<0.001
Unclear	13	−0.28 [−0.8281; 0.2604]		92%	<0.001
High	2	−2.14 [−28.0772; −23.7942]		98%	<0.001

SMD, *standardized mean difference*; LBW, *low birth weight*; NBW, *normal birth weight*; CI, *confidence interval*; I^2^, *heterogeneity*.

^1^Cianfarani et al. [36], Evagelidou et al. [37], Ibáñez et al. [38], Ibáñez et al. [39], Jaquet et al. [40], Mohamed et al. [41], Takaya et al. [42], Terrazzan et al. [43], Wang et al. [24].

**Fig 2:**
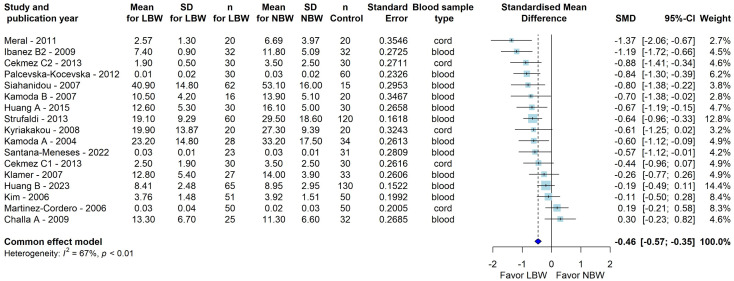
The association between circulating adiponectin levels and low birth weight as compared to normal birth weight controls after the exclusion of outlier studies. Forest plot comparing mean circulating adiponectin levels in LBW subjects and NBW controls after outlier removal. The overall effect size is SMD = −0.46 μg/ml [95% CI: −0.57; −0.35], P < 0.0001. The blue diamond represents the overall effect size and confidence interval. Heterogeneity is estimated at I^2^ = 67% (P < 0.01), assessed using Cochran’s Q and I^2^ statistics. LBW, *low birth weight;* NBW, *normal birth weight;* SMD, *standardized mean difference;* SD, *standard deviation;* CI, *confidence interval;* I^2^, *heterogeneity.* Seventeen data points are presented for 16 studies, as Ibäñez et al. [38] included two LBW groups compared to one NBW group.

An influence analysis was conducted to determine whether the presence of possible outliers influenced the results, and with an additional GOSH Plot analysis, nine studies were identified as primary contributors to the overall heterogeneity. Following the exclusion of outliers [24,36–43], the meta-analysis of the remaining 16 studies still showed significantly lower adiponectin levels in LBW individuals as compared to NBW (SMD = −0.46 μg/ml [95% CI: −0.57; −0.35], P < 0.0001) with moderate heterogeneity (I^2 ^= 67%, P < 0.01) (**Fig 2**). Notably, the dataset comprising 24 studies yielded 26 data points, as two studies each contributed with two separate data points [38,44]. One of these studies had one data points identified as an outlier and thus excluded, while the other data point remained in the analysis [38]. Therefore, although nine outlier studies were excluded, the final analysis included 16 studies.

The extended meta-analysis consisting of 14 studies comparing adiponectin levels between HBW and NBW subjects failed to establish significant evidence of a difference (SMD = −0.36 μg/ml [95% CI: −1.06; 0.33], P = 0.27; I^2 ^= 95%) and therefore no further assessments were made regarding the role of HBW.

### Subgroup analysis assessing the impact of postnatal growth

**Fig 3:**
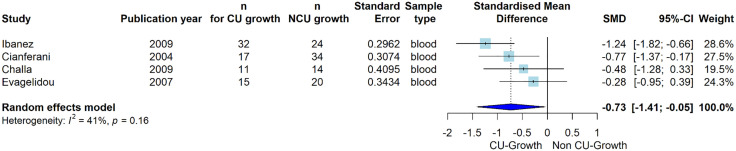
The association between circulating adiponectin levels and catch-up growth vs. non-catch-up growth. Forest plot comparing mean circulating adiponectin levels in CU-growth versus non-CU-growth groups across four studies. The overall effect size is SMD = −0.73 [95% CI: −1.41; −0.05], P = 0.04. The blue diamond represents the overall effect size and confidence interval. Heterogeneity is estimated at I^2^ = 41.3% (P = 0.16) assessed using Cochran’s Q and I^2^ statistics. SMD, *standardized mean difference;* SD, *standard deviation;* CI, *confidence interval;* I^2^, *heterogeneity;* CU, *catch-up growth.* Catch-up growth is defined as either corrected height SDS ≥ 0 [36,37,44] or matched height, weight, and BMI to NBW controls [38].

To investigate the postnatal growth patterns, a subgroup analysis was performed to ascertain whether the occurrence of catch-up growth (CU-growth) among LBW individuals influenced adiponectin levels later in life. Out of all the studies included, four specifically explored this phenomenon, revealing compelling evidence of decreased adiponectin levels in LBW individuals who underwent CU-growth compared to those who did not (SMD = −0.73 [95% CI: −1.41; −0.05], P = 0.04) (**Fig 3**).

### Sensitivity analysis and meta regression

Sensitivity analysis of age categories, sample type, and risk of bias was conducted to determine whether these factors had any influence on the high heterogeneity. The finding of lower circulating adiponectin levels in LBW subjects tended to be more robust in neonates compared to older individuals with also the heterogeneity being lowest within this group (SMD = −0.52, I^2 ^= 67%, P = 0.55). Studies with low risk of bias showed a stronger correlation between low adiponectin and LBW but were not statistically significant (SMD = −0.48, I^2 ^= 63%, P = 0.70) (**Table 4**). In addition, a meta-regression was performed with the same moderators investigated in the sensitivity analysis with the addition of publication year to examine possible influence. Neither of the moderators, including age category, sample type, risk of bias, or publication year had any significant impact on the results.

**Table 4:** SMD and associated heterogeneity in a sensitivity analysis of 16 studies comparing adiponectin levels in LBW individuals with NBW controls (excluding outliers^1^).

Characteristics	Data points	Summary SMD (95% CI)	P-value for effect interaction	I^2^ (95% CI)	P-value for heterogeneity
Age categories					
Neonates	9	−0.52 [−0.6946; −0.3506]	0.55	67%	<0.001
Infants	1	−0.56 [−1.1162; −0.0150]		–	–
Children	7	−0.40 [−0.5580; −0.2445]		74%	<0.001
Blood sample type					
Cord	5	−0.43 [−0.6650; −0.1984]	0.77	80%	<0.001
Blood	12	−0.46 [−0.5996; −0.3403]		62%	<0.001
Risk of bias					
Low	7	−0.48 [−0.6388; −0.3277]	0.70	63%	<0.001
Unclear	9	−0.45 [−0.6388; −0.3277]		72%	<0.001
High	1	−0.25 [−0.7665; 0.2551]		–	–

SMD, *standardized mean difference*; LBW, *low birth weight*; NBW, *normal birth weight*; CI, *confidence interval;* I^2^, *heterogeneity*.

^1^Cianfarani et al. [36], Evagelidou et al. [37], Ibáñez et al. [38], Ibáñez et al. [39], Jaquet et al. [40], Mohamed et al. [41], Takaya et al. [42], Terrazzan et al. [43], Wang et al. [24].

### Publication bias and funnel plot

**Fig 4:**
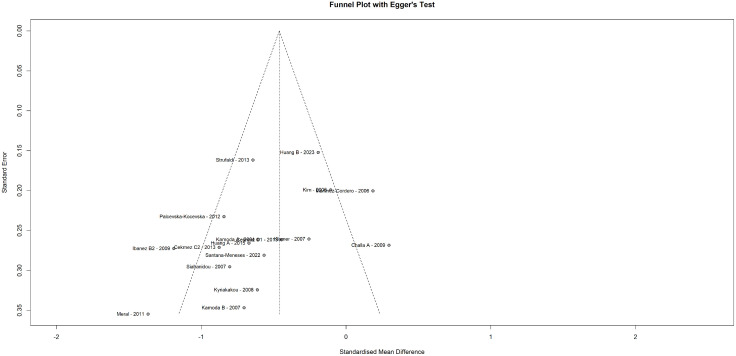
Funnel plot with Egger’s test. Funnel plot with Egger’s Test was performed to evaluate the publication bias.

Publication bias was assessed through visual inspection of the funnel plot illustrating slight asymmetry (**Fig 4**). However, Egger’s test did not indicate the presence of publication bias (P = 0.07).

### Strength and direction of the association between adiponectin and birth weight

**Fig 5:**
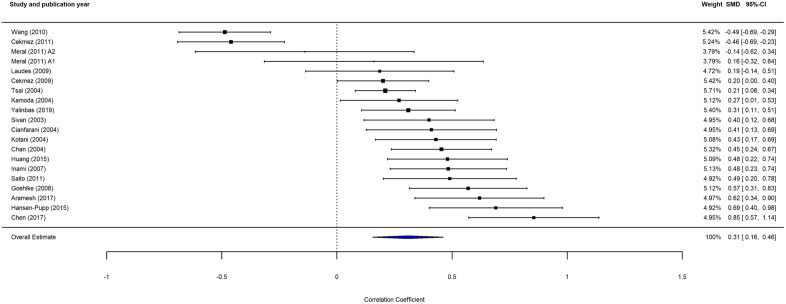
Analysis of pooled Pearson correlations from 19 studies examining the relationship between adiponectin levels and birth weight. Forest plot showing the correlation between circulating adiponectin levels and birth weight across 19 studies. The pooled estimate is r = 0.31. Heterogeneity is estimated at I^2^ = 89% (P < 0.0001), assessed using Cochran’s Q and I^2^ statistics. Pearson correlations (r) were extracted from eligible studies, and 95% CIs were calculated using the random effects model. SMD, *standardized mean difference;* CI, *confidence interval;* I^2^, *heterogeneity.* Twenty data points are presented for 19 studies, as Meral et al. [38] provide a correlation coefficient for both small and large for gestational age.

The relationship between adiponectin levels and birth weight was further explored by inferential statistics. The provided Pearson correlations from 19 studies were included for analysis using a random-effects model (**Fig 5**). The pooled overall estimate of 0.31 ([95% CI: 0.16; 0.46], P < 0.0001) indicates a moderate but positive correlation between adiponectin and birth weight, suggesting a linear relationship between the two variables. Substantial heterogeneity was observed (I^2 ^= 89%, P < 0.0001), though no further analyses were made to assess variability.

## Discussion

This systematic review provides firm evidence of lower circulating adiponectin levels in a categorical analysis of individuals born with LBW in comparison to NBW, which was further supported by a statistically significant positive correlation between circulating adiponectin levels and birth weight. The results all together support the notion of reduced circulating adiponectin levels as a potential key mediator of increased cardiometabolic disease risk in people born with LBW.

The result from the meta-analysis comes with moderate heterogeneity after the exclusion of the outliers, which were based on their relative effect sizes, defined as either extremely small or large, thereby disproportionally influencing the overall effect size and heterogeneity, potentially compromising the study’s reliability. While some heterogeneity is expected in meta-analyses, excluding outliers that disproportionately influence the heterogeneity helps preserve the robustness of the findings without altering the overall results of the meta-analysis. Sensitivity and meta-regression analyses did not identify any moderators that could fully account for the remaining heterogeneity. However, differences in ethnicity, geographic location, assay type, and study settings are the most likely contributors to the seen variability. The heterogeneity can to some extent be attributed to variation in study design as the meta-analysis encompasses cross-sectional, cohort, and case-control studies. Therefore, the diversity in study design, along with the unexplored variables, inherently contributes to the remaining heterogeneity. Additionally, differences in study populations and/or designs among outliers may further contribute to the heterogeneity. For instance, some studies included LBW children with catch-up growth [39], short LBW children [38], or preterm neonates [43], and when visually examining the funnel plot, these outliers appeared to be linked with publication bias. However, Egger’s test did not detect significant bias, suggesting the asymmetry is more likely to be due to heterogeneity rather than bias. Notably, the asymmetry was subsequently eliminated after excluding the outliers, which did not alter the overall result of the meta-analysis. Furthermore, it is important to point out that the excluded outliers also found lower levels of adiponectin in LBW individuals [36,39,40,42,43], indicating that their exclusion was not based on their findings, but rather because of their attribution to the high heterogeneity (S7 Fig). An exception to this is the study by Evagelidou et al. [37], which did not find lower serum adiponectin levels in a cohort of pre-pubertal LBW children. Interestingly, the authors interpreted their negative findings as indicative of a transient adaptation mechanism involving a compensatory elevated adiponectin release to counteract peripubertal insulin resistance, further speculating that a subsequent decrease in adiponectin levels may follow later in life in these individuals. In support of this, Jaquet et al. [40] reported a U-shaped relationship between serum adiponectin levels and insulin resistance in young adults with LBW, which consistent with Evagelidou et al., was interpreted to indicate a compensatory and somewhat disproportional increase in circulating adiponectin levels in LBW during the peripubertal period. This, in turn, could also be explained by some kind of transient adiponectin resistance in LBW adolescents [40]. While further studies are needed to understand these observations in LBW adolescents, they do not change the overall interpretation of the current study, providing firm evidence of reduced circulating adiponectin levels in LBW subjects.

Besides the puberty-associated changes, our analysis furthermore indicates a relatively stronger correlation between LBW and adiponectin levels in early life, subsequently diminishing with aging (**Tables 3** and **4**). This trend aligns with prior studies showing a positive correlation between body weight and circulating adiponectin levels during infancy, which is replaced by an inverse relationship in adulthood [45–47]. Indeed, the observed changes in the associations with birth weight over time might partly be explained by this shift. These associations are further influenced and complicated by the notion that LBW subjects retain lower body weight, adult height, and even to some extent, lower BMI throughout life [48–51]. To this end, LBW subjects tend to accumulate excessive visceral and hepatic fat [8,39], which in turn may represent ectopic fat deposition associated with immature and epigenetically mediated changes in subcutaneous adipose tissue cells [9,52]. While further studies are needed to delineate the causal inferences underlying these observations, the current results support the notion of a potentially important role of adiponectin in mediating the increased risk of cardiometabolic disease among people born with LBW.

LBW infants and children often experience postnatal CU-growth [53], which in turn has been associated with a further increased risk of adult cardiometabolic diseases [54,55]. Notably, our subgroup analysis of the four studies that addressed this topic indicated that CU-growth in LBW is associated with further reduced circulating adiponectin levels compared with LBW individuals who did not experience CU-growth [36–38,56] (**Fig 3**). This suggests that reduced adiponectin levels may not only be causally linked to increased cardiometabolic disease risk in LBW individuals per se but that a further distinct reduction in adiponectin is observed in those experiencing CU-growth, placing them at an even higher risk. Nevertheless, the causal inference related to this finding needs to be substantiated by mechanistic as well as prospective studies, and particularly it must be determined whether the further reduction in adiponectin levels is a direct contributor to cardiometabolic risk or a secondary consequence of the excess adiposity characterizing LBW CU-growth individuals.

**Fig 6:**
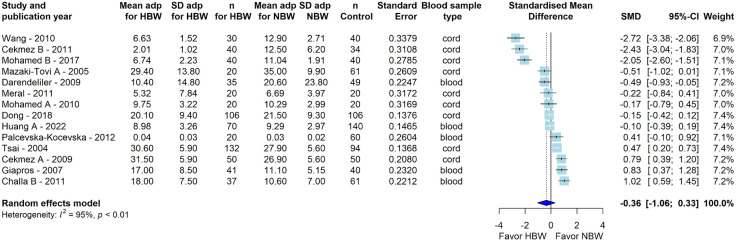
The association between circulating adiponectin levels and high birth weight as compared to normal birth weight controls. Forest plot comparing mean circulating adiponectin levels in HBW versus NBW groups. The overall effect size is SMD = −0.36 μg/ml [95% CI: −1.06; 0.33], P = 0.27. The blue diamond represents the overall effect size and confidence interval. Heterogeneity is estimated at I^2^ = 95% (P < 0.01), assessed by Cochran’s Q and I^2^ statistics. NBW, *normal birth weight;* HBW, *high birth weight;* SMD, *standardized mean difference;* SD, *standard deviation;* CI, *confidence interval;* I^2^, *heterogeneity.*

In addition to LBW, HBW has also been associated with an increased risk of adult cardiometabolic diseases, which in turn may be related to and/or explained by maternal hyperglycemia in pregnancy. Indeed, some – but not all – studies have reported a U- or J-shaped relationship between birth weight and risk of T2D [4, 57,58]. In the current meta-analysis, we found an overall positive correlation between adiponectin levels and birth weight across the entire spectrum (**Fig 5**), with the association being further strengthened by a z-value of 3.95. This finding of a high z-value indicates that the observed correlation coefficient is 3.95 standard errors away from zero, suggesting a highly significant result caused by a consistent statistical pattern within the data. However, with some non-significant trend towards relatively lower adiponectin levels among HBW individuals (**Fig 6**), further studies are needed to understand the extent to which reduced circulating adiponectin levels may be implicated in mediating the association between HBW and the risk of cardiometabolic diseases.

### Strengths and limitations

To the best of our knowledge, this is the first meta-analysis investigating the association between adiponectin levels and birth weight across various age groups. Our findings of reduced adiponectin levels in LBW individuals remain robust across all sensitivity and subgroup analyses, including in- or exclusion of outliers. The absence of comparable meta-analyses presents both a strength and a limitation. As the first study to systematically synthesize evidence across different age groups, we offer a thorough understanding of how fetal programming may influence the development of T2D and cardiovascular disease. However, the lack of similar meta-analyses limits direct validation. Nevertheless, our results align with smaller yet high-quality deep phenotyping studies comparing LBW and NBW individuals [8,13], which, despite their relevance, were not included in our meta-analysis due to misalignment with our inclusion criteria.

As for potential limitations in our study, we applied varying definitions of LBW, equating it with SGA, potentially contributing to observed heterogeneity. This decision was made to adhere to the definitions applied by the authors of the original studies. In support of this approach, it was recently argued that LBW and SGA should be encompassed under one category, as these infants are all adversely affected by prenatal exposures leading to some level of alteration in their fetal programming [17]. Additionally, the sensitivity analyses were limited by the smaller sample size available for older age groups. Furthermore, adiponectin exists in low as well as in a high molecular form, with the high molecular form being proposed to have the biggest impact in vivo [45,59]. However, due to limited information available from the applied assays [45], we were unable to determine the impact of the molecular forms of adiponectin in the different studies. Despite this limitation, our findings remain robust, as adiponectin – regardless of its molecular form – is considered to improve insulin sensitivity. Nevertheless, there is a need for large clinical and prospective studies interrogating the extent to which the different molecular forms of adiponectin may be involved in differential physiological or pathophysiological mechanisms, and/or potentially associated with differential cardiometabolic outcomes or disease manifestations.

Our finding of a robust association between LBW and lower circulating adiponectin levels is clinically relevant as it raises the possibility of using adiponectin as a biomarker to determine cardiometabolic disease risk later in life in LBW subjects, as well as subjects with unknown, normal, or high birth weight. However, larger prospective studies are needed to determine the sensitivity, including false positive and negative predictions, and to benchmark it against other clinical or biomarker predictors.

Moreover, our findings could have implications for strategies aimed at improving prenatal and nutritional care. As LBW is a recognized risk factor for the later development of T2D [1–4], potentially mediated by lower adiponectin levels, interventions focused on enhancing maternal nutrition and optimizing prenatal care may play a vital role in mitigating this risk. With the addition of adiponectin’s potential as a biomarker for risk monitoring after birth, targeted prevention of T2D in LBW subjects may include both antenatal and postnatal prevention and risk mitigation efforts. Additionally, longitudinal studies exploring adiponectin across different life stages in LBW individuals could help identify critical periods when adiponectin levels are most influential, uncovering opportunities for targeted interventions at key developmental stages.

Finally, although the exclusion of studies due to language restriction is a potential limitation, only three studies were excluded for this reason. The large number of included studies with high-quality data minimizes the likelihood of a significant impact on the overall findings. Furthermore, since most relevant studies in this field are published in English, the risk of systematic bias from language restriction is likely to be minimal.

## Conclusion

In conclusion, this current meta-analysis provides firm evidence that individuals born with LBW exhibit decreased levels of adiponectin, potentially contributing to their elevated risk of T2D. These findings highlight the potential of adiponectin as an early biomarker of metabolic dysfunction and cardiometabolic risk in LBW individuals, offering a valuable tool in clinical practice for the prevention of T2D.

## Supporting information

S1 Table Search strategy. (DOCX)

S2 Table Studies written in languages not understood by the review team for consideration in future analysis by fluent readers. (DOCX)

S1 Fig Risk of bias assessment for case-control studies. (DOCX)

S2 Fig Risk of bias assessment for cohort studies. (DOCX)

S3 Fig Risk of bias assessment for cross-sectional studies. (DOCX)

S4 Fig Relationship between age groups and adiponectin levels. (DOCX)

S5 Fig Influence of blood sample type on adiponectin levels. (DOCX)

S6 Fig Impact of risk of bias on adiponectin levels. (DOCX)

S7 Fig Analysis of the excluded outliers comparing adiponectin levels in LBW and NBW subjects. (DOCX)

S1 Checklist PRISMA Checklist. (DOCX)
