# Supplementary material for: Is low birth weight associated with lower adiponectin levels? - A systematic review and meta-analysis
Source: PLoS One. 2025 Dec 2;20(12):e0335598. doi: 10.1371/journal.pone.0335598 (PMC12671802; doi:10.1371/journal.pone.0335598)
Supplement: S1 Table — (DOCX) [file pone.0335598.s001.docx]

**Supplementary data**

**Table S1. Search strategy**

Ovid MEDLINE(R) all (1946 to July 4th, 2024).

| **#** | **Searches** | **Results** |
| --- | --- | --- |
| 1 | Receptors, Adiponectin/ | 1280 |
| 2 | exp Adipokines/ | 42669 |
| 3 | (adiponectin$ or adipokin$ or adipocytokin$).ti,ab,kf,kw. | 33753 |
| 4 | or/1-3 | 57895 |
| 5 | exp Infant, Low Birth Weight/ | 39093 |
| 6 | Fetal Blood/ | 32054 |
| 7 | (((small* or little) adj2 gestational age) or (impaired fetal adj (growth or development)) or ((fetal or intra uterine or intrauterine) adj (growth restrict$ or growth retard$)) or LBW or IUGR or birth weight or birthweight or "weight at birth" or "weight at time of birth" or cord blood or fetal blood).ti,ab,kf,kw. | 145164 |
| 8 | or/5-7 | 166826 |
| 9 | and/4,8 | 1276 |
| 10 | exp Animals/ not Humans/ | 5158236 |
| 11 | 9 not 10 | 1096 |

Embase**(**1974 to 2024 July 4th).

| **#** | **Searches** | **Results** |
| --- | --- | --- |
| 1 | adiponectin/ or adiponectin receptor/ or adiponectin receptor 1/ or adiponectin receptor 2/ | 43989 |
| 2 | adipocytokine/ or adipocytokine receptor/ | 18498 |
| 3 | (adiponectin$ or adipokin$ or adipocytokin$).ti,ab,kf,kw. | 50511 |
| 4 | or/1-3 | 62395 |
| 5 | exp low birth weight/ | 60724 |
| 6 | fetus blood/ | 8493 |
| 7 | (((small* or little) adj2 gestational age) or (impaired fetal adj (growth or development)) or ((fetal or intra uterine or intrauterine) adj (growth restrict$ or growth retard$)) or LBW or IUGR or birth weight or birthweight or "weight at birth" or "weight at time of birth" or cord blood or fetal blood).ti,ab,kf,kw. | 204881 |
| 8 | or/5-7 | 222771 |
| 9 | and/4,8 | 1086 |
| 10 | animal experiment/ not (human experiment/ or human/) | 2567406 |
| 11 | 9 not 10 | 1013 |
| 12 | limit 11 to exclude medline journals | 113 |

| Search | (((TI=(adiponectin* or adipokin* or adipocytokine*)) OR (AB=(adiponectin* or adipokin* or adipocytokine*)) OR (AK=(adiponectin* or adipokin* or adipocytokine*))) AND ((TI=(small for gestational age or little gestational age or impaired fetal growth or impaired fetal development or fetal growth restrict* or fetal growth retard* or intra uterine growth restrict* or intra uterine growth retard* or intrauterine growth restrict* or intrauterine growth retard* or LBW or IUGR or birth weight or birthweight or "weight at birth" or "weight at time of birth" or cord blood or fetal blood)) OR (AB=(small for gestational age or little gestational age or impaired fetal growth or impaired fetal development or fetal growth restrict* or fetal growth retard* or intra uterine growth restrict* or intra uterine growth retard* or intrauterine growth restrict* or intrauterine growth retard* or LBW or IUGR or birth weight or birthweight or "weight at birth" or "weight at time of birth" or cord blood or fetal blood)) OR (AK=(small for gestational age or little gestational age or impaired fetal growth or impaired fetal development or fetal growth restrict* or fetal growth retard* or intra uterine growth restrict* or intra uterine growth retard* or intrauterine growth restrict* or intrauterine growth retard* or LBW or IUGR or birth weight or birthweight or "weight at birth" or "weight at time of birth" or cord blood or fetal blood)))) |
| --- | --- |
| Date run | July 4^th^, 2024 |
| Results | 878 |
| Database | Web of Science Core Collection |
| Entitlements | WOS.IC: 1993 to 2023, WOS.CCR: 1985 to 2023, WOS.SCI: 1900 to 2023, WOS.AHCI: 1975 to 2023, WOS.BHCI: 2005 to 2023, WOS.BSCI: 2005 to 2023, WOS.ESCI: 2005 to 2023, WOS.ISTP: 1990 to 2023, WOS.SSCI: 1956 to 2023, WOS.ISSHP: 1990 to 2023 |

Web of Science search strategy (v0.1)
