# Supplementary material for: Is low birth weight associated with lower adiponectin levels? - A systematic review and meta-analysis
Source: PLoS One. 2025 Dec 2;20(12):e0335598. doi: 10.1371/journal.pone.0335598 (PMC12671802; doi:10.1371/journal.pone.0335598)
Supplement: S2 Table — (DOCX) [file pone.0335598.s002.docx]

**Supplementary data**

**Table S2.** **Studies written in languages not understood by the review team for consideration in future analysis by fluent readers**

| Reference |
| --- |
| Purnomowati (2012). [Differences of Plasma Adiponectine, Asymmetric Dimethylarginine and Brachial Artery Vasodilatation Response in Young Adult with Low and Normal Birth Weight History]. |
| Strocchio (2007). [Changes in circulating levels of adiponectin and leptin in children during the first two years of life]. |
| Yamamoto (2009). [Analysis of serum adiponectin and leptin in mothers and umbilical cord blood]. |
