# Supplementary material for: Is low birth weight associated with lower adiponectin levels? - A systematic review and meta-analysis
Source: PLoS One. 2025 Dec 2;20(12):e0335598. doi: 10.1371/journal.pone.0335598 (PMC12671802; doi:10.1371/journal.pone.0335598)
Supplement: S2 Fig — (DOCX) [file pone.0335598.s004.docx]

**Supplementary data**

**Figure S2. Risk of bias assessment of cohort studies**

**
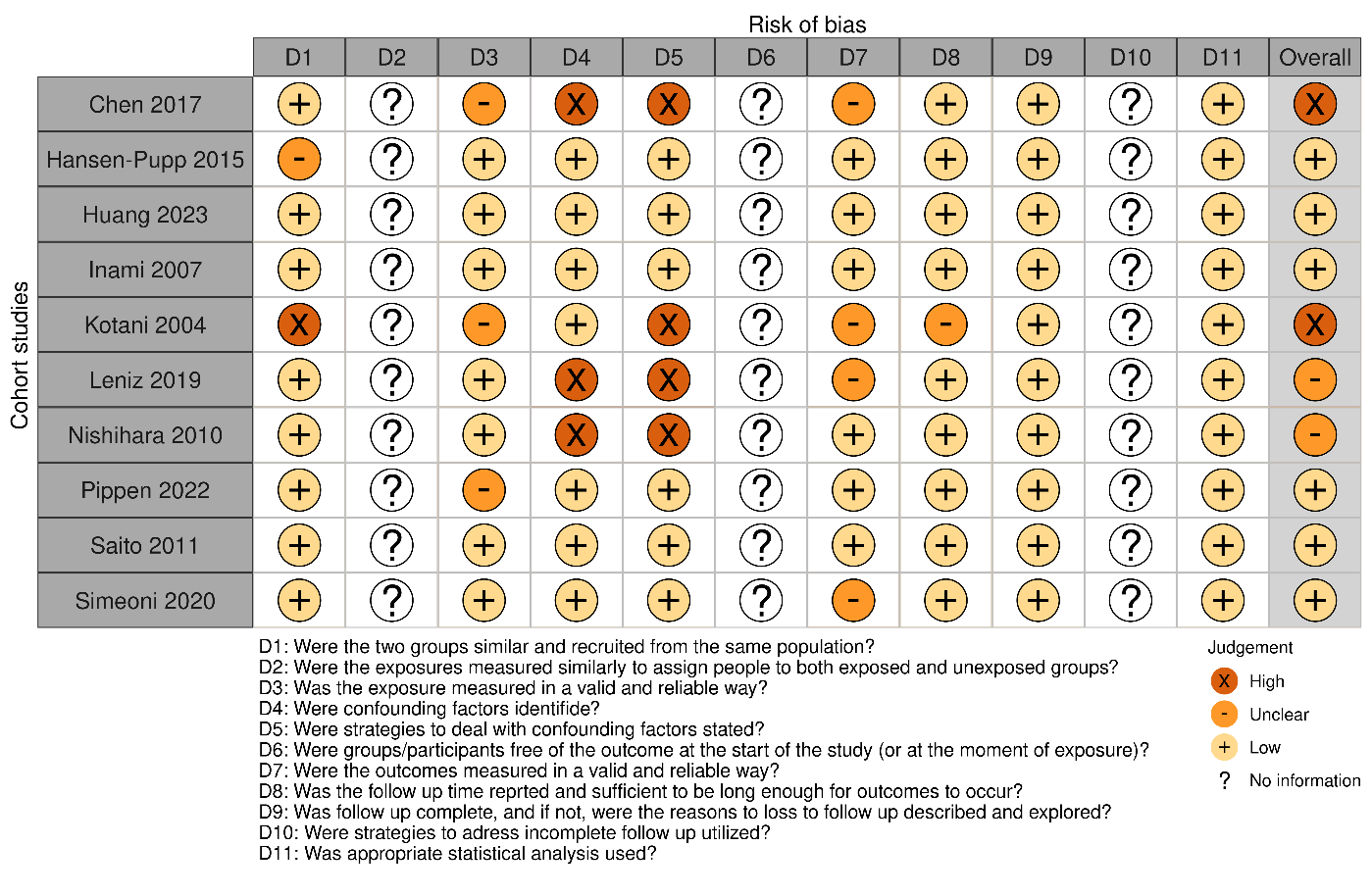
**

This table presents the risk of bias assessment for the included cohort studies. Author names and publication years are listed on the left, while the top of the table features a series of questions labeled as “D” followed by a number. Each question evaluates whether the study reports the relevant information. Based on the overall judgement for each question, a cumulative score is assigned to each study in the right panel. Color coding is utilized to indicate the level of bias: yellow (+) signifies low risk of bias, orange (-) indicates unclear risk of bias, red (X) represents high risk of bias, and ? indicates no information.
