# Supplementary material for: Is low birth weight associated with lower adiponectin levels? - A systematic review and meta-analysis
Source: PLoS One. 2025 Dec 2;20(12):e0335598. doi: 10.1371/journal.pone.0335598 (PMC12671802; doi:10.1371/journal.pone.0335598)
Supplement: S4 Fig — (DOCX) [file pone.0335598.s006.docx]

**Supplementary data**

**Fig S4. Relationship between age groups and adiponectin levels**

**
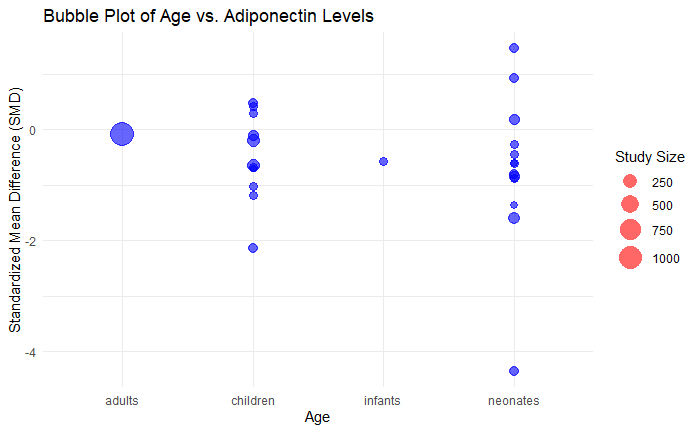
**

This bubble plot visualizes the association between age categories (adults, children, infants, and neonates) and standardized mean differences (SMD) in adiponectin levels. Each bubble represents a study, with its position indicating the SMD and its size corresponding to the study sample size. The color-coded legend on the right denotes study sizes in participants. The plot reveals that adiponectin levels in adults are relatively stable around an SMD of 0, suggesting minimal differences in levels. In contrast, children and neonates exhibit greater variability in SMD values, with some studies indicating lower adiponectin levels compared to controls. The results suggest that age may influence adiponectin levels, although not of statistical significance.
