# Supplementary material for: Is low birth weight associated with lower adiponectin levels? - A systematic review and meta-analysis
Source: PLoS One. 2025 Dec 2;20(12):e0335598. doi: 10.1371/journal.pone.0335598 (PMC12671802; doi:10.1371/journal.pone.0335598)
Supplement: S5 Fig — (DOCX) [file pone.0335598.s007.docx]

**Supplementary data**

**Fig S5. Influence of blood sample type on adiponectin levels**

**
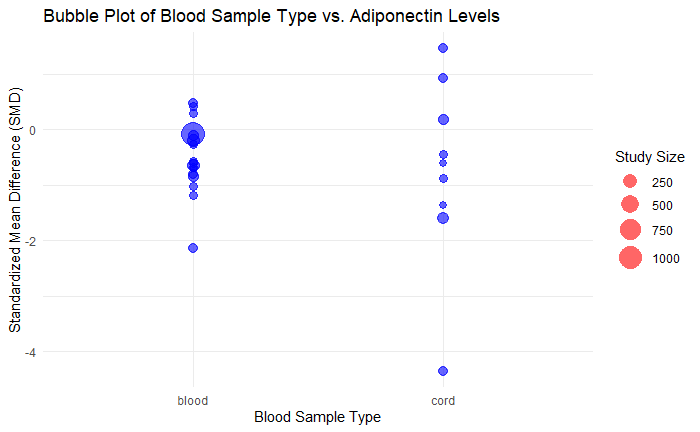
**

This bubble plot presents the relationship between blood sample type (venous blood and cord blood) and standardized mean difference (SMD) in adiponectin levels in studies examining low birth weight (LBW). Each bubble represents a study, with its position indicating the SMD and its size corresponding to the study sample size. The color-coded legend on the right denotes study sizes in participants. The plot shows that studies using venous blood samples exhibit a broader range of SMD values, with some studies reporting a more negative association between LBW and adiponectin levels. In contrast, studies using cord blood samples tend to cluster around a narrower range, suggesting more consistent effect sizes. Additionally, the larger study weights appear more concentrated within the venous blood group, indicating that these studies using venous blood may contribute more heavily to the overall effect estimate. This pattern suggests that the choice of blood sample type could influence the observed adiponectin levels. However, in this case, this is not statistically significant.
