# Supplementary material for: Is low birth weight associated with lower adiponectin levels? - A systematic review and meta-analysis
Source: PLoS One. 2025 Dec 2;20(12):e0335598. doi: 10.1371/journal.pone.0335598 (PMC12671802; doi:10.1371/journal.pone.0335598)
Supplement: S6 Fig — (DOCX) [file pone.0335598.s008.docx]

**Supplementary data**

**Fig S6. Impact of risk of bias on adiponectin levels**

**
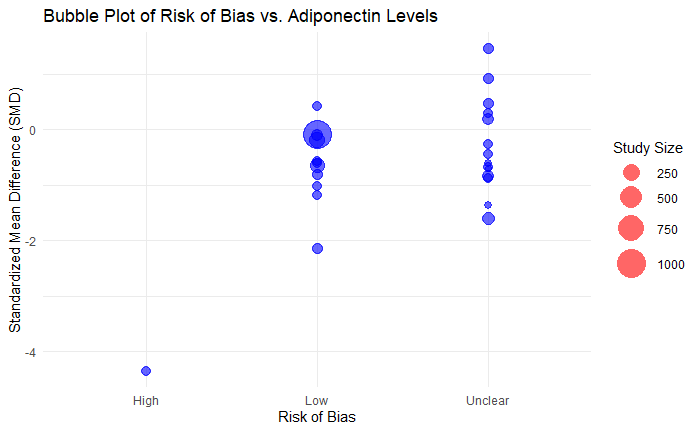
**

This bubble plot demonstrates the association between risk of bias (categorized as high, unclear, or low) and standardized mean differences (SMD) in adiponectin levels in studies on low birth weight (LBW). Each bubble represents a study, with its position indicating the SMD and its size corresponding to the study sample size. The color-coded legend on the right denotes study sizes in participants**.** The plot reveals that the studies with low risk of bias show a relatively concentrated distribution of effect sizes, mostly clustering around zero, suggesting more consistency in their findings. In contrast, studies categorized as having an unclear or high risk of bias display a wider range of effect size, with some outliers reporting stronger negative associations. These variations may indicate that studies with greater methodological uncertainties yield more heterogeneous results, potentially influencing the overall interpretation of the results. The studies with a larger weight are predominantly in the low-risk category, further reinforcing the reliability of these findings.
