# Supplementary material for: Is low birth weight associated with lower adiponectin levels? - A systematic review and meta-analysis
Source: PLoS One. 2025 Dec 2;20(12):e0335598. doi: 10.1371/journal.pone.0335598 (PMC12671802; doi:10.1371/journal.pone.0335598)
Supplement: S7 Fig — (DOCX) [file pone.0335598.s009.docx]

**Supplementary data**

**Fig S7. Analysis of the excluded outliers comparing adiponectin levels in LBW and NBW subject**


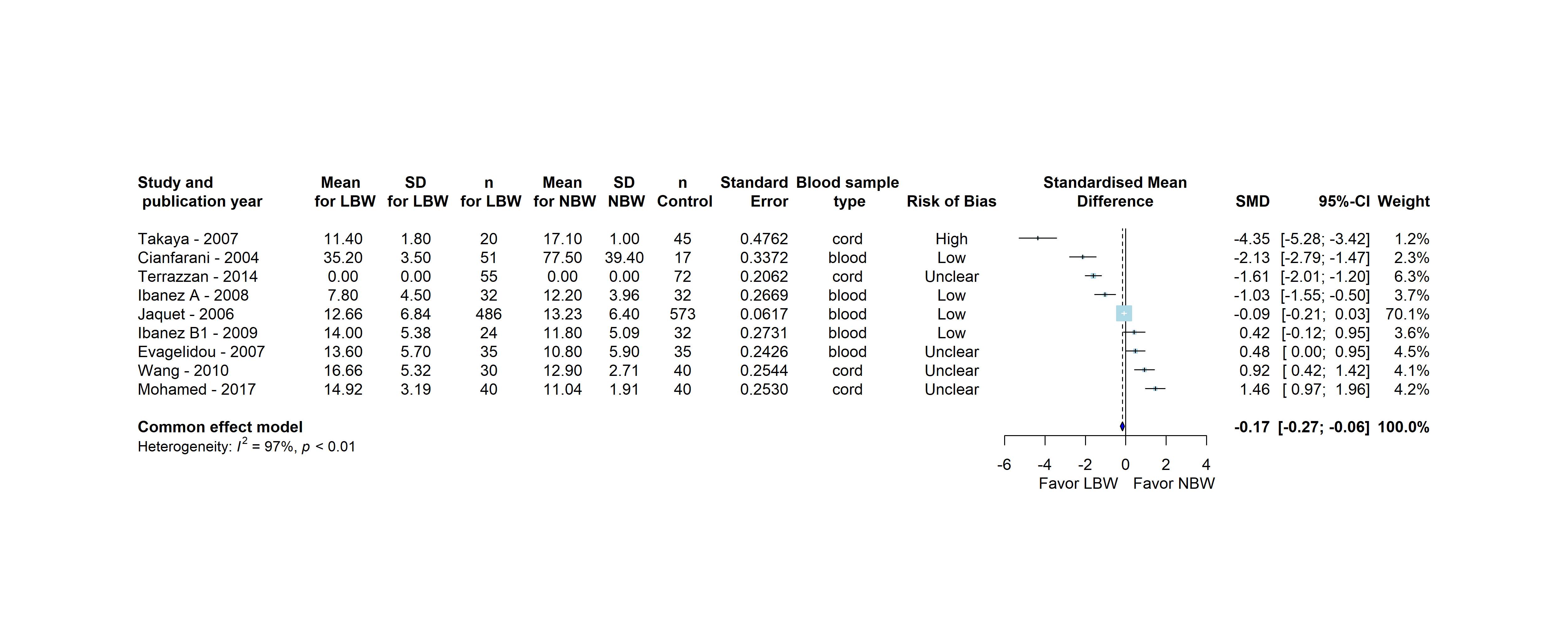


Forest plot comparing mean circulating adiponectin levels in LBW with NBW controls of the excluded outlier studies. The overall effect size (SMD = -0.17 μg/ml [95%-CI: -0.27; -0.06], p<0.0001) indicates that adiponectin levels are still significantly lower in LBW individuals as compared to NBW controls. The diamond (blue) represents the overall effect size and confidence interval. Heterogeneity (I^2^ = 97%, p<0.01) was assessed through Cochran’s Q and I^2^ statistics.

*LBW, low birthweight; NBW, normal birthweight; SMD, standardized mean difference; SD, standard deviation; CI, confidence interval; I^2^, heterogeneity*.
